# Hypothesis – buttressed rings assemble, clamp, and release SNAREpins for synaptic transmission

**DOI:** 10.1002/1873-3468.12874

**Published:** 2017-10-31

**Authors:** James E. Rothman, Shyam S. Krishnakumar, Kirill Grushin, Frederic Pincet

**Affiliations:** ^1^ Department of Cell Biology Yale University School of Medicine New Haven CT USA; ^2^ Laboratoire de Physique Statistique Ecole Normale Supérieure PSL Research University Université Paris Diderot Sorbonne Paris Cité Sorbonne Universités UPMC Univ CNRS Paris France

**Keywords:** membrane fusion, SNARE, synaptic transmission

## Abstract

Neural networks are optimized to detect temporal coincidence on the millisecond timescale. Here, we offer a synthetic hypothesis based on recent structural insights into SNAREs and the C2 domain proteins to explain how synaptic transmission can keep this pace. We suggest that an outer ring of up to six curved Munc13 ‘MUN’ domains transiently anchored to the plasma membrane via its flanking domains surrounds a stable inner ring comprised of synaptotagmin C2 domains to serve as a work‐bench on which SNAREpins are templated. This ‘buttressed‐ring hypothesis’ affords straightforward answers to many principal and long‐standing questions concerning how SNAREpins can be assembled, clamped, and then released synchronously with an action potential.

## Abbreviations

**CPX**, complexin

**DOPC**, dioleoylphosphatidylcholine

**DOPE**, dioleoylphosphatidylethanolamine

**PC**, phosphatidylcholine

**PE**, phosphatidylethanolamine

**PM**, plasma membrane

**PS**, phosphatidylserine

**SV**, synaptic vesicles

**Syt**, synaptotagmin

The human brain is privileged to draw far more than its share of energy from the body to provide us the advantage of rapid and efficient information processing. This is achieved primarily on the basis of the principle of coincidence detection. Patterns closely and consistently correlated in time are likely to be causally related and therefore predictive. To maximize coincidence detection, neural circuits are optimized for synchronicity, which they achieve on the low millisecond timescale. Synchronicity is ultimately limited only by the combined speeds of action potentials and of synaptic transmission. These in turn rely on the biophysics of ion channel opening/closing and of vesicle fusion. While we have an excellent understanding of the former, how synaptic vesicles (SV) at nerve endings can release their neurotransmitters by membrane fusion within a millisecond remains a major unanswered question.

The mystery here stems from the fact that the SNARE proteins that drive membrane fusion intrinsically operate on a timescale of about a second. While this speed is more than ample to support the majority of the physiological processes, like cell division and hormone release, it is 10^3^–10^4^ too slow to enable synchronous neurotransmitter release. Evidently the SNAREs are somehow specially organized at synapses to achieve this remarkable feat. While we have a ‘parts list’ of the additional proteins that co‐operate with SNAREs, there is presently no coherent understanding of how they assemble and operate together.

Here, we formulate the ‘buttressed‐ring’ hypothesis based on recent advances in the structures of component parts. This speculative model affords a natural explanation for how these proteins can co‐operate in a symmetrical structure function far faster and synchronously than they could individually. It is consistent with and can explain a wide range of physiology and suggests many novel experiments according to its specific requirements and predictions.

As background, decades of research combining biochemistry, genetics, and neurophysiology, the list of components required for action‐potential‐triggered secretion of neurotransmitters at neuronal synapses is clear 1, 2. These include the SNARE proteins that physically mediate fusion of transmitter‐containing SV with the presynaptic plasma membrane (PM); the soluble chaperone‐like proteins Munc18 and Munc13 that function to organize and facilitate the initial assembly of the SNARE proteins between the vesicle and PM; the SV protein synaptotagmin (Syt) that triggers fusion when it binds Ca^2+^, and the generally soluble protein complexin (Cpx) that suppresses spontaneous (un‐signaled) release while also potentiating evoked synaptic transmission 1, 2, 3, 4.

We know that SV that are ready to release at the presynaptic PM (‘readily releasable pool’) are closely bound by SNAREpins that are incompletely zippered 5, 6, 7. To reach this stage, several important steps are needed. First, SV are captured at specialized ‘active zones’ by the elongated RIM tethering protein, along with Munc13 binding to the SV's Rab GTPase proteins 3, 8. Then, the membrane‐distal C2B domain of SV‐localized Syt (15–20 copies per SV) binds to the PM‐specific phosphoinositide PIP2, bringing the SV and PM within molecular contact distance 9, 10, 11. This enables the partial assembly between the v‐SNARE VAMP (also termed Synaptobrevin; ~ 70 copies per SV) and the t‐SNARE subunits Syntaxin and SNAP‐25, both emanating from the PM 1, 9, 12.

Many questions remain concerning how SNAREpin assembly is initiated. It is known that syntaxin enters (separately from SNAP‐25) as a 1 : 1 complex with Munc18 that is initially concentrated at the cytoplasmic surface of the presynaptic PM in ~ 75 nm diameter nano‐domains rich in PIP2 8, 10, 13. SNAP‐25 is anchored to the inner surface of the PM by several covalently linked fatty acid chains 14. In a poorly understood but likely coupled series of reactions requiring Munc18 and Munc13, Munc18 is displaced from one of its binding sites on the SNARE complex forming helix of Syntaxin 8, 15, 16, which is then combined with the corresponding helical segments of VAMP and SNAP‐25 to form an ~ 8‐nm‐long four helix bundle that is assembled about 2/3rds of the way to completion.

What prevents further assembly (termed ‘zippering’) – and therefore immediate fusion – is key but its structural basis is unknown. Such partly assembled SNAREpins must somehow be stabilized (‘clamped’) from further assembly despite that completion is intrinsically strongly favorable (~ 35 *k*_B_ *T*) and occurs spontaneously in the absence of other proteins 17, 18, 19. Instead, the SV awaits the entry of Ca^2+^ from outside the cell (resulting from action‐potential‐triggered opening of nearby voltage‐gated Ca^2+^ channels) into the cytoplasm where resting Ca^2+^ is low (~ 100 nm) 20. Syt and Cpx somehow co‐operate to create this clamp, but it is not known how this occurs 3. When Ca^2+^ binds to Syt, the clamp is removed, and the vesicle can now fuse, presumably as the SNAREs complete their zippering into a complete four helix bundle 4. How Syt transduces this signal to SNAREpins is likewise still unclear despite many biochemical and structural studies.

With this in mind, we call attention to several new facts that have very recently emerged which, if they are relevant physiologically, can combine to enable a coherent framework that can fill in many of the gaps in our understanding of the molecular mechanisms of synaptic transmission.

**Figure 1:**
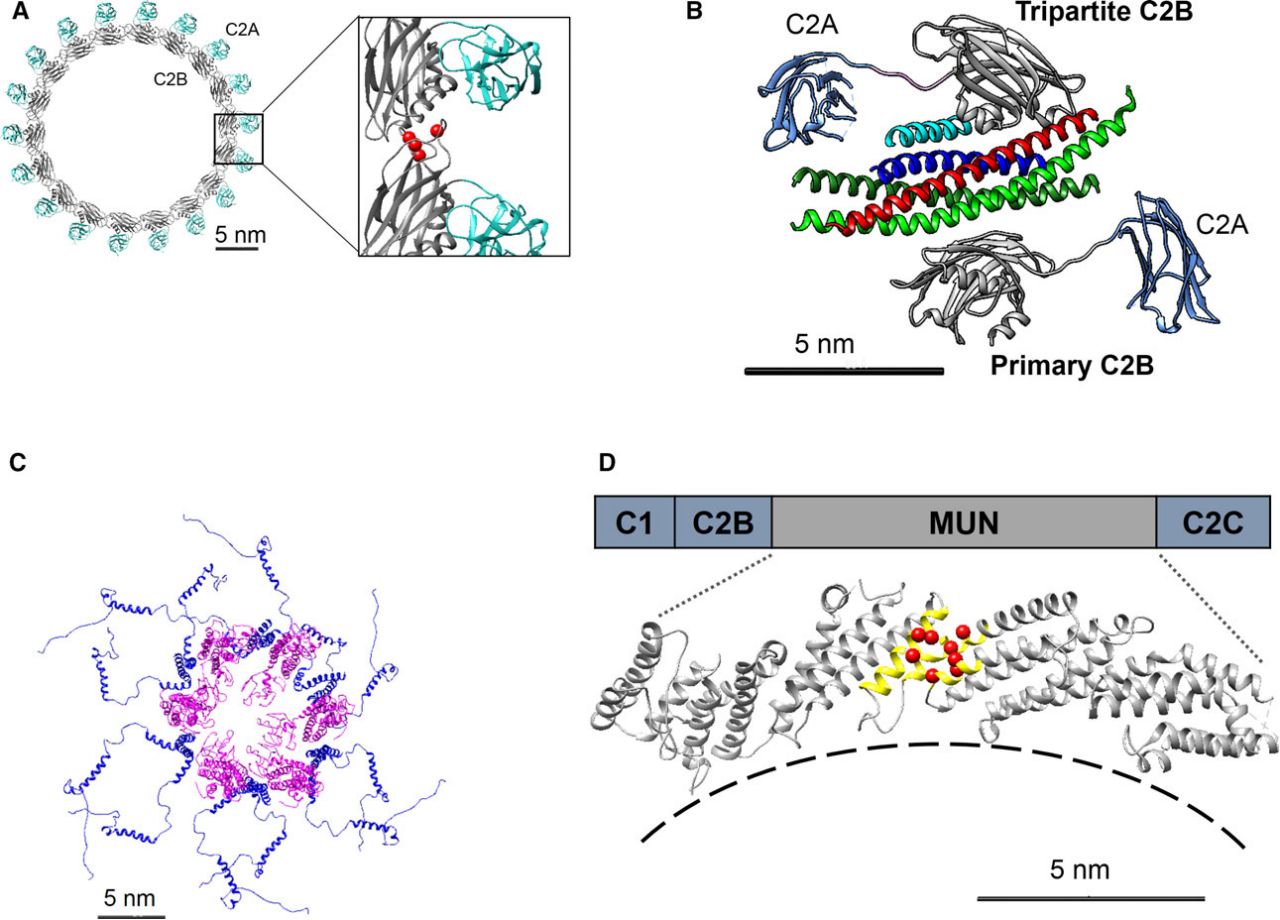
Recent structural insights into the protein machinery involved in synaptic transmission. (A) 3D‐reconstruction of the ring‐like oligomers formed by Syt1, the primary Ca^2+^ sensor for neurotransmitter release at the synapse 21, 22, 23. These oligomers, which are typically 20–35 nm in diameter (12–20 copies), assemble based on the interactions between the C2B domains (gray), with the C2A (cyan) domain locating outside the ring structure. The Ca^2+^ binding loops (red dots) are involved in oligomer formation and are located within the C2B‐C2B interfaces and thus render the oligomers sensitive to Ca^2+^. (B) Crystal structure of a prefusion SNARE‐Cpx‐Syt1 complex revealed that two Syt1 molecules can bind to opposite sides of the same SNAREpin, each via its C2B domain 24. One Cpx‐independent (termed ‘primary’) interface involves contacts with both SNAP25 helices (green); the other Cpx‐dependent interface (termed ‘tripartite’) involves contacts with helices derived from Cpx (cyan), syntaxin (red), and VAMP2 (blue). (C) Electron microscopy‐derived structure of a purified synaptophysin‐VAMP2 complex reveals a hexameric ring architecture wherein six synaptophysin (magenta) molecules bind and organize six VAMP2 (blue) dimers such that they are directionally oriented toward the target membrane 26. The ring is stabilized by contacts among transmembrane helices within the bilayer. (D) The domain arrangement of Munc13‐1 protein, which involves C1, C2B, and C2C domain flanking its widely conserved MUN domain. The amino terminal C2A domain is not shown. The crystal structure of the Munc13‐1 MUN domain consists of an elongated, arch‐shaped structure formed by α‐helical bundles, with a highly conserved hydrophobic pocket approximately in the middle (yellow highlights) 27. Mutations in this region (residues in red) compromise Munc13 ability to chaperone SNARE assembly 28, 29. Figure adapted from Ref. 24, 26, 27.

First, isolated Syt1 was discovered to polymerize into 20–35 nm diameter planar ring‐like oligomers 21, 22, 23 containing 12–20 copies based on interactions between the C2B domains (Fig. 1A). Polymerization is triggered by ATP at cytoplasmic concentrations or by binding to PIP2, which is found in the PM but not the SV. Rings formed on acidic lipid surfaces are stable with Mg^2+^, but dissociate when Ca^2+^ is added 21, 23. Upon Ca^2+^ binding, the aliphatic loops flanking the C2B domain's Ca^2+^ binding sites insert into the membrane bilayer as Ca^2+^ bridges the key aspartic acids and bilayer phosphatidylserine (PS). The ring dissociates because the same interface that is used to form the ring is also used to interface with the bilayer, and both cannot happen at the same time 21.

Second, a very recent and important high‐resolution crystal structure from the laboratories of Brunger and Sudhof 24 shows how monomeric Syt is bound to a partially zippered SNARE complex along with Cpx (Fig 1B), building on an earlier structure assembled without Cpx 25. It shows the unexpected recruitment of two monomeric C2B domains – derived from two distinct Syt molecules – to opposite surfaces of the SNAREpin. One site (‘primary’) is Cpx‐independent and involves both helices of SNAP‐25. The second Cpx‐dependent (‘tripartite’) site involves different contacts between a distinct C2B unit and portions of the helices of Cpx, Syntaxin, and VAMP.

Third, electron microscopic analysis (Fig. 1C) has revealed that the v‐SNARE VAMP is pre‐organized within the SV into hexameric units by interactions of its transmembrane domain with that of the multi‐spanning SV membrane protein synaptophysin 26.

Finally, a very recent and important high‐resolution structure of the complete core functional domain of Munc13 (the MUN domain) from the laboratories of Rizo and Ma 27, 28 reveals a rigid, curved, planar shape (Fig 1D), building on their earlier, less complete structures 29.

Here, we show how these structures elegantly combine to form a hexagonally symmetrical assembly between the membranes that naturally explains how SNAREpins can be stably clamped and yet very rapidly and synchronously released. We explain how our novel ‘buttressed‐ring’ hypothesis can thus provide important missing links between SNARE assembly clamping, and release, while also making many specific and testable predictions as well as raising numerous questions for future research.

## Inner and outer rings to template SNAREpin assembly

The MUN domain of Munc13 is the functional unit required for SNARE assembly both *in vivo* 30, 31, 32, 33 and in cell‐free systems 15, 16, 28. Although it is not specifically known, it is simplest to assume that one MUN domain catalyzes the assembly of only one SNAREpin at a time. Mutations preventing SNARE binding and/or assembly localize to a surface approximately in the middle of the MUN domain 28, suggesting that this is the main active site. MUN is made up of four subdomains which are revealed in X‐ray structures 27, 28 to consist of analogous globular helix‐based units. The MUN domain overall has a rigid, planar curved shape ~ 15 nm in contour length (Fig. 1D). It has been well‐established that MUN functions as a tether between SV and PM 34, 35, but if this were the only function of the MUN domain, it would not explain its curved structure; nor would it locate the presumed active site (yellow region in Fig. 1D) near the PM where it is needed to catalyze SNAREpin assembly.

**Figure 2:**
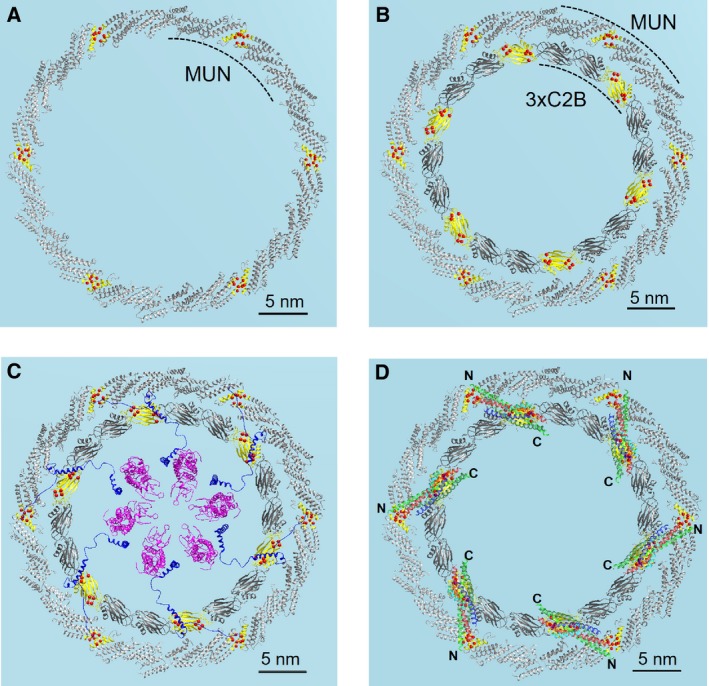
Buttressed rings of Munc13 and Syt are proposed to act as work stations for SNAREpin assembly, clamping, and release. (A) Proposed hexameric end‐to‐end organization of MUN domain of Munc13 to form a flat, ring‐like structure. The view from the top with the PM (blue) below is shown. (B) The hypothetical MUN domain ring closely encloses a Syt ring‐oligomer formed by 18 Syt C2B domains. This concentric ring organization naturally aligns the hydrophobic pocket in the middle of MUN domain with the primary interface of every third Syt1 C2B (both shown in yellow highlights). (C) When the SV (not shown for clarity) approaches the PM, the hexagonally arranged VAMPs (blue) organized by synaptophysin (magenta) are suitably positioned to reach the proposed active surface of each MUN subunit to template the SNARE assembly. (D) As a result, a total of six SNAREpins are proposed to be assembled spanning the outer and inner rings with their carboxy‐terminal transmembrane domains (marked by ‘C’) inside the rings inserted above and below into SV and PM, respectively.

**Figure A1:**
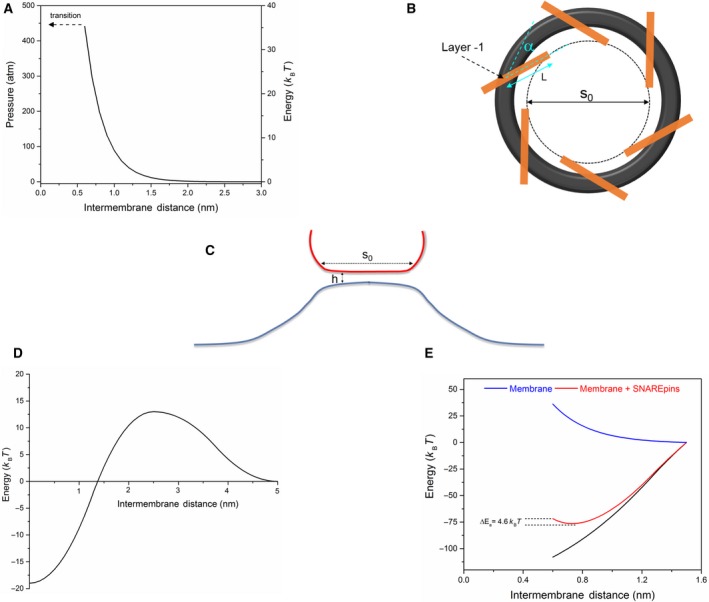
(A) Short range repulsion between two DOPC/DOPE (60/40) lipid bilayers. The values are based on published data [47–49]. On the right axis is plotted the corresponding energy when the pressure is applied over a 13 nm² area, i.e. a 4 nm disk. Fusion occurs when the pressure reaches 440 atm, or equivalently 35 *k*_B_ *T*. (B) The ring model as viewed from top. Six SNAREpins are uniformly distributed over the C2B inner ring. The inner diameter of the ring is 19 nm and the outer diameter 25 nm. Layer −1 of the SNAREpin is located right at the mid‐point (22 nm diameter) of the ring. The SNAREpin is positioned at an angle α = 40° to the tangent of the ring and the inner extension between layer −1 and the end of the SNARE domain (layer +8) is at *L* = 7 nm. Layer +8 of the six SNAREpins are located on a circle with a diameter *s*_0_ = 17 nm as calculated in the text of the Appendix. (C) Side view. The two membranes (SV in red and PM in blue) are pulled in towards each other by the SNAREpins. At equilibrium, the inter‐membrane distance, defined as the water layer thickness, is *h*. (D) Energy landscape corresponding to the process of zippering of the last several layers of the SNAREpin. Only the values of the minima and the maximum have been reported [18]; their relative positions are unknown. Here, we assume this process occurs over 5 nm and that it is harmonic. The shape of the contour was chosen to achieve continuity in energy and force at the midpoint between two consecutive extremes. Note that this includes the zippering of the C‐terminal linker domains, immediately after the SNARE domains. The inter‐membrane distance is assumed equal to 0 when the linker domains are fully zippered. (E) Zoom‐in view of the short‐range repulsion energy (panel A) and the SNAREpin zippering for six SNAREpins (6× panel D) between the transition distance (0.6 nm) and 1.5 nm with energy values at 1.5 nm for clarity. The sum of the two curves provides the actual energy landscape produced by the SNAREpins and the short‐range repulsion. An intermediate state appears at 0.7 nm and the activation energy to transit from this intermediate state to the fused state is 4.6 *k*_B_ *T*.

**Figure A2:**
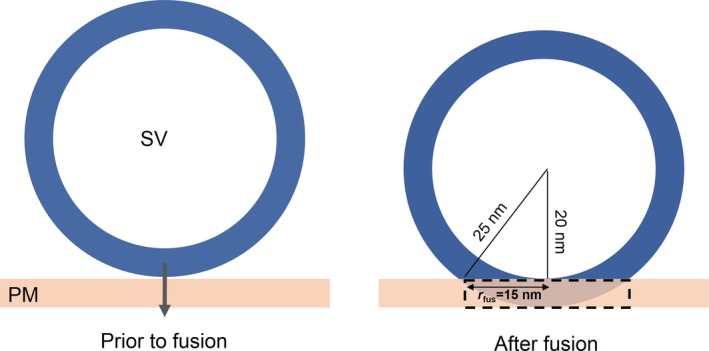
Relative position of the synaptic vesicle (SV) and the plasma membrane (PM) prior to fusion (left). Upon the action of the SNAREpins, the synaptic vesicle moves towards (arrow) and coalesces with the plasma membrane. The positions after fusion are shown at right. The overlapping regions have mixed. Because they are up to 1000 times more viscous than water, the slowest movement comes from the displacement of lipids themselves. Hence, upon the collective action of SNAREpins, the limiting factor in the diffusion‐limited fusion process is the movement of the synaptic vesicle over the thickness of a bilayer (5 nm). The radius of the impacted region is *r*_fus_ = 15 nm. The black box represents the contour of the disk over which the calculation of the viscous force is made (see text).

We note that the ~ 15‐nm arc subtended by each planar MUN domain (Fig. 1D) curves ~ 60˚. As a consequence, six MUN domains placed end to end can be readily arranged to form a flat, closed ring (Fig 2A). Remarkably, such a hypothetical MUN domain‐based hexameric ring would closely enclose (Fig. 2B) an inner ring of 18 Syt C2B domains based on the Syt ring structure (Fig. 1A). These two rings are not only concentric but they are also co‐planar (see Fig. A1 for alternate views and space‐filling representations). To place the MUN domains in the same plane as the Syt ring, and to enable their expected binding to PM lipids, the C1/C2B and C2C units which flank either end of the MUN domain would all need to be the located radially outside the proposed MUN domain‐based ring (see Fig. A2). While other arrangements are possible, for example, in which one or both C2 domains are included in the inner ring, these arrangements would not allow the flanking C1 or C2 domains to bind the PM where they are known to interact with diglyceride and PIP2, respectively 36, 37, 38, 39. Additionally, there is a powerful evolutionary reason to favor an outer ring that excludes the C2 domains: most MUN‐domain containing proteins possess neither C1 nor C2 domains and yet they template SNARE assembly for constitutive vesicle fusion. Therefore, a general mechanism for the conserved MUN domain cannot involve C1 or C2 in its core structure.

**Figure A3:**
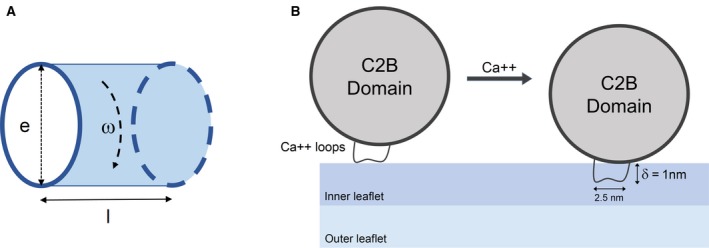
Movements of a C2B domain after its detachment from the ring: (A) C2B monomer is modeled as a cylinder of diameter *e* = 5 nm and length *l* = 5 nm that rotates around its axis with an angular velocity ω. The direction of the axis is dictated by SNAREpin zippering, which orients the movement. (B) Loop insertion. The aliphatic loops flanking that Ca^2+^ binding site, located just above the cytoplasmic leaflet of the PM, are modeled as inserting by moving 1 nm down vertically into the PM after Ca^2+^ binds. The shape of the loops is reasonably modeled as a cylinder 1 nm long and 2.5 nm in diameter.

The proposed concentric ring organization naturally aligns every third C2B domain in the inner Syt ring with the middle portion of each of the six MUN domains (yellow highlights in Fig. 2B), so that if each MUN domain assembles one SNAREpin, the entire ring would assemble six of them spaced evenly around. When an ~ 35‐nm diameter SV approaches these concentric rings on the PM, its hexagonally positioned VAMPs (templated by mixed hexamer with Synaptophysin in the SV) fit well within the inner ring, in such a way that the N‐terminal portion of VAMP (which has a high propensity for helix formation 40 and assembles first) can reach the proposed active surface (yellow) of each MUN subunit at every third Syt C2B (Fig. 2C and A3).

Based on these geometrical relationships, and the need for the product SNAREpins to be immediately captured in one or another way to prevent further zippering and thereby clamp fusion, we hypothesize that the outer ring of up to 6 MUN domains and the inner ring of ideally 18 vesicle‐derived Syt C2B domains co‐operate to act as up six ‘workstations’ that simultaneously template up to six half‐zippered SNAREpins, drawing a VAMP from each vertex of its hexagon on the SV and combining it with one Syntaxin‐Munc18 and one SNAP‐25 drawn from the PM. Recent functional reconstitution data provide strong support for the idea of a combined Syt‐C2B/Munc13 surface for assembling SNAREpins 41 and suggest that the primary binding site of C2B plays a templating role with respect to SNAP‐25 assembly.

**Figure A4:**
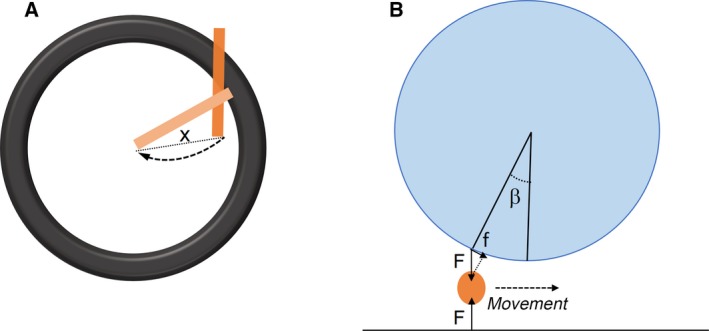
(A) Top view. A SNAREpin is released from the ring. Initially it was positioned on the ring (grey). As terminal zippering proceeds, the SNAREpin moves so as to position itself radially with its transmembrane domains (inserted above into the SV and below into the PM) towards the center of the ring (light orange). During the process, the movement of the transmembrane domain is approximated by a straight line, wherein *x* is the displacement from the initial position. (B) Side view (perpendicular to the SNAREpin). The contribution of the zippering force, *F*, to the movement corresponds to its projection tangentially to the SV,* f*.

Combining these new ideas (Fig. 2D) suggests that up to six SNARE complexes initiated in proximity to every third Syt C2B domain in the inner ring are juxtaposed to a catalytic portion of a MUN unit in the outer ring (Fig. A4). This would result in SNAREpins symmetrically anchored in the opposing bilayers within the inner Syt ring, providing the precise topology required for fusion to result upon completion of zippering toward the membranes.

## Clamping of the SNAREpins

**Figure 3:**
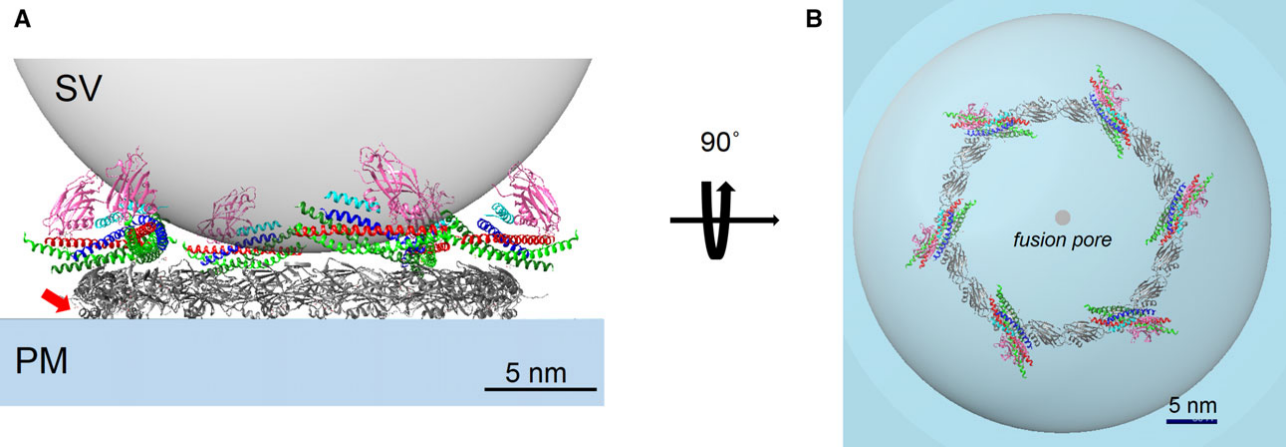
Clamping of SNAREpin terminal zippering by radial retention according to the buttressed‐ring hypothesis. The assembling SNAREpins are retained on the inner Syt ring via the ‘primary’ SNARE‐C2B domain interaction. In the ring oligomers, the second independent C2B domain (magenta) sits above the SNAREpin bound via the ‘tripartite’ binding site in conjunction with Cpx. Such an arrangement would allow the ‘tripartite’ C2B to bind the SV membrane, likely via lipid interaction. As a result, each SNAREpin is held in a vice‐like clamp between the two C2B domains, each bound to and buttressed by the opposing membranes preventing further zippering. Cpx likely strengths this clamp by additionally anchoring its SNAREpin to the SV membrane to which it is bound via its C‐terminal domain (not shown). Note that in the ring arrangement, the conserved helical extension in Syt C2B (red arrow), which is the basis of tripartite binding faces the PM. Both the side view (A) and the top view (B) are shown. Note: for clarity, the outer ring of Munc13 is not shown.

Completion of SNAREpin zippering must be delayed to enable the release of neurotransmitters to be triggered by the arrival of an action potential at the nerve ending (signaled by Ca^2+^ entry). The recent Syt‐Cpx‐SNARE structure 24, when combined with the Syt ring structure, lends a likely explanation as to how this could occur. We assume that each of the six SNAREpins will be retained locally by the nearest Syt C2B in the inner ring in the same geometry found in the crystal structure by the ‘primary’ 24, 25 binding site involving the two helices of SNAP‐25 but not VAMP or Syntaxin (Fig. 3: A side view, B top view). In this arrangement, the second, independent ‘tripartite’ 24 C2B domain (shown in magenta in Fig 3), bound via a structurally conserved helical domain to the opposite side of the SNAREpin (involving VAMP, Syntaxin, and CPX), sits above the SNAREpin immediately juxtaposed to the SV membrane. This orients the polybasic region of the tripartite C2B and its Ca^2+^/Mg^2+^ binding surface so that one or the other could interact with the negatively charged PS on the surface of the SV. Note that the tripartite C2B could not engage in ring assembly in this orientation since polymerization involves the same Ca^2+^/Mg^2+^ binding surface 21, 23. Note also that the helical extension of Syt C2B which engages with Cpx, VAMP, and Syntaxin in the tripartite interaction contacts the PM (red arrow in Fig. 3A) when the C2B is in the inner ring, unavailable for tripartite binding.

The result is a three‐layered protein structure in which a middle layer of SNAREpins is firmly sandwiched between two ‘clamping’ layers of C2 domains: a bottom (PM‐bound) ring of primary Syt C2Bs; and a top layer of tripartite (SV‐bound) C2Bs. This arrangement would trap each SNAREpin in a vice‐like grip between the two membranes, held both from above (SV) and from below (PM). This grip would be further strengthened by zippering itself, which produces a substantial force that pulls the two membranes toward each other 17, 19. The top layer of tripartite C2Bs buttresses the lower layers using the SV as an anchor. Cpx likely adds to this buttress because it links from the SNAREpin to the SV where it is anchored by its SV‐binding curvature‐sensitive C‐terminal domain 42, 43, also explaining why altering or removing this domain from Cpx results in a reduction of clamping 42, 44.

**Figure 4:**
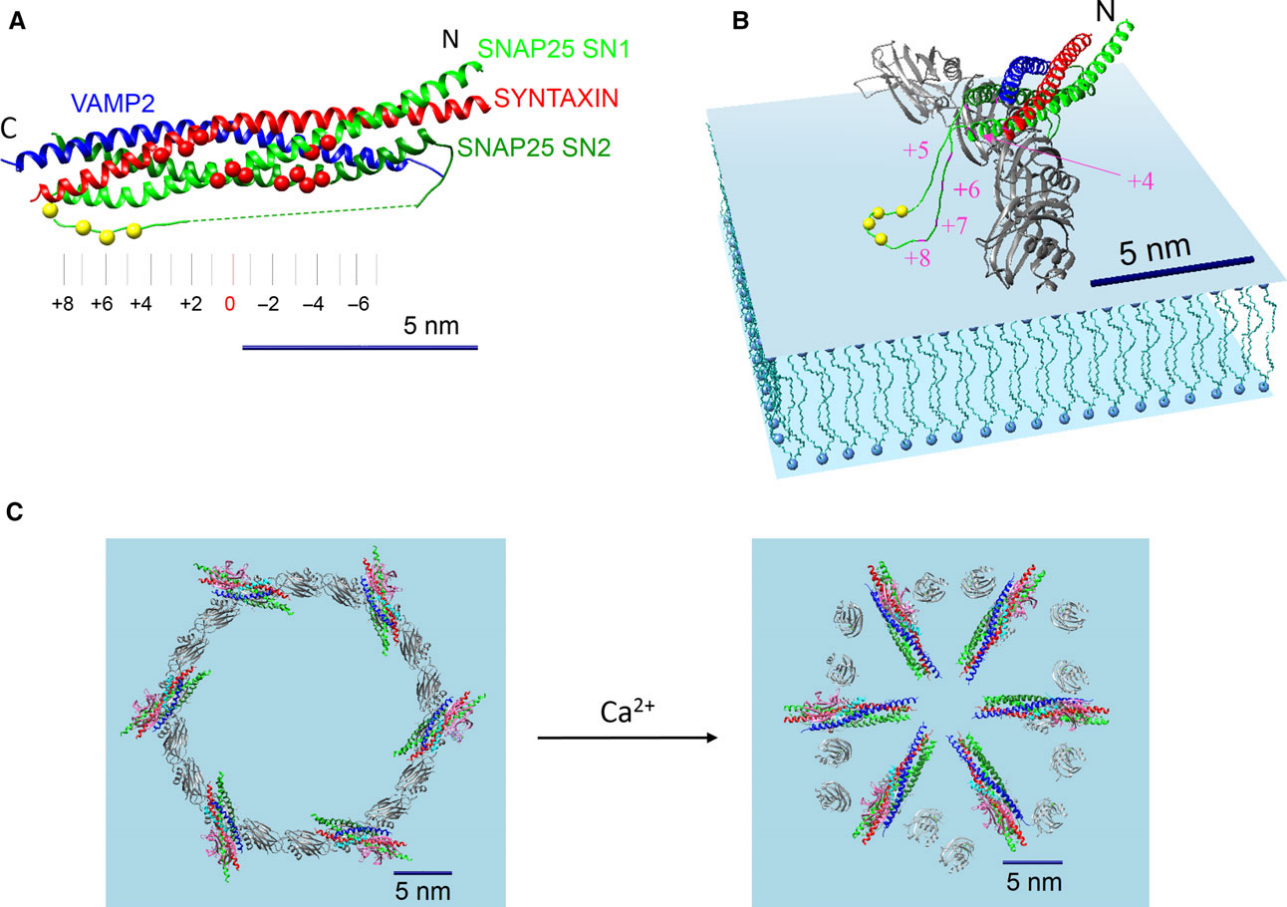
Clamping of SNAREpin terminal zippering by enforced spatial separation according to the buttressed‐ring hypothesis. (A) Terminology to describe the anatomy of the fully assembled SNARE complex four‐helix bundle 45. The hydrophobic layers (alternately consisting of 3 and 4 residues each) are numbered from −7 to +8. The locations of the residues contacting Syt1 C2B in the ‘primary’ Syt binding site (red dots) suggest that the SNAREpins have to be zippered up to or beyond the +3 layer to accommodate Syt binding. (B) Modeling shows that given the elevated positioning of the SNAREpin atop the Syt ring, the residues of the SNAP25 SN1 helix that assemble into layers +5 to +8 (red spots) to complete zippering and trigger fusion would instead need to be nearly fully extended in this geometry to enable the adjacent Cys palmitoylation sites (yellow dots) to be inserted into the PM bilayer (blue). This suggests that zippering beyond layer +4 will be impeded due to the spatial separation between the membranes imposed by the ring. (C) The SNAREpins on the Syt ring are held at an angle such that even if they were to fully zipper their four helix bundles to layer +8, the tips of these bundles would be positioned inside the ring too far out radially (~ 17 nm from +8 tip to +8 tip) from the center to enable fusion (see Appendix 1 for details). Thus, the Syt ring also radially restrains the full zippering of the SNARE complex. Upon the influx of Ca^2+^, the Syt ring oligomers are disrupted as Syt molecules rotate to insert into the PM. This frees the SNAREpins to complete zippering and move in radially to open the initial fusion pore.

SNAREpin binding to a primary site on the Syt ring requires the SNAREpin's four helix SNARE bundle to be ~ 2/3rds zippered (to at least layer +4) because the primary site on the SNAREpin side contains sequences running from layer −5 to layer +1 in SNAP‐25 and layer +4 in Syntaxin 24, 25. The anatomy of the four helix bundle, the location of the primary Syt binding site, and the ‘layer’ nomenclature 45 are shown in Fig. 4A for the reader's convenience. Completing fusion requires the full four helix bundle to fully assemble, up to layer +8. For the proposed clamp to prevent fusion, it, therefore, must prevent zippering up to layer +8.

As mentioned above, when SNAREpins complete zippering they concomitantly pull the membranes together, and geometry requires that the SNAREpins themselves move in radially toward the central contact point around which the fusion pore will open. Therefore, in principle, the SNAREpins can be prevented from further zippering and triggering fusion by either: (a) holding the membranes too far apart or (b) preventing the SNAREpins from moving radially inward in the membrane plane, or both. Our proposed structure does both.

First, the height of the ring plus the SNAREpin atop it maintains the two bilayers too far apart (~ 4 nm; see Fig. 3A) to enable them to fuse since bilayer fusion requires an approach of ~ 1 nm or less 46, 47, 48, 49. This was the central idea of an earlier version of this hypothesis termed the ‘washer model’ 21, 23. In addition, due to the manner in which SNAP‐25 is firmly anchored at the PM by covalently attached lipids, the height of the inner ring also creates a SNARE complex‐specific steric constraint to prevent further zippering of this helix (Fig. 4B). SNAP‐25 is multiply palmitylated at the Cys residues within the polypeptide loop that links its two SNARE helices, SN1 and SN2 (Fig. 4A), and this covalent attachment has been shown to be critical in neuroendocrine cells models 50, 51. Given the thickness of the ring, modeling (Fig. 4B) shows that the hydrophobic layers +5 to +8 of SNAP‐25 SN1 helix (colored in red) must be nearly fully extended for the palmitylated Cys residues (colored yellow) to reach the PM bilayer, suggesting that zippering beyond layer +4 will be impeded due to the separation imposed by the ring. The c‐terminal zippering could be additionally blocked by Cpx as its accessory helix in the tripartite binding mode is ideally positioned to create a steric block to impede SNARE assembly 52, 53, 54.

Second, concerning the radial restraint mechanism, the SNAREpins on the ring are oriented more circumferentially than radially (Fig. 4C). This positions the membrane‐proximal end of the fully zippered four helix bundle (thru layer +8) too far out radially (~ 8.5 nm from the center) to enable fusion, which is expected to result only when the SNAREpins have moved in radially to about 2 nm from the center (Appendix 1) to open an initial fusion pore^55^, ^56^, ^57^, ^58^. Note that the SNAREpins may be further restrained by interactions with the MUN domains in the outer ring. In sum, the ring should synergistically enforce a clamp on the SNAREpins according to all of the above mechanisms.

## Release of the SNAREpins

Reversing the clamp requires disassembly of the Syt C2B ring, which occurs when it binds to Ca^2+^ ions 21, 23. Importantly, the same surface of Syt C2B that interacts to form the ring in the absence of Ca^2+^ interacts in a different (~ 90° rotated) geometry with the PM when it binds Ca^2+^ 21, 23. Soluble Syt rings do not disassemble when Ca^2+^ is added; they only do so when bound to acidic phospholipid surfaces 22. The difference is that now the Ca^2+^ binding loops can sandwich Ca^2+^ with membrane PS and insert the flanking aliphatic residues, gaining ~ 21 *k*_B_ *T* in energy^59^, tipping the balance toward disassembly of the ring.

**Figure A5:**
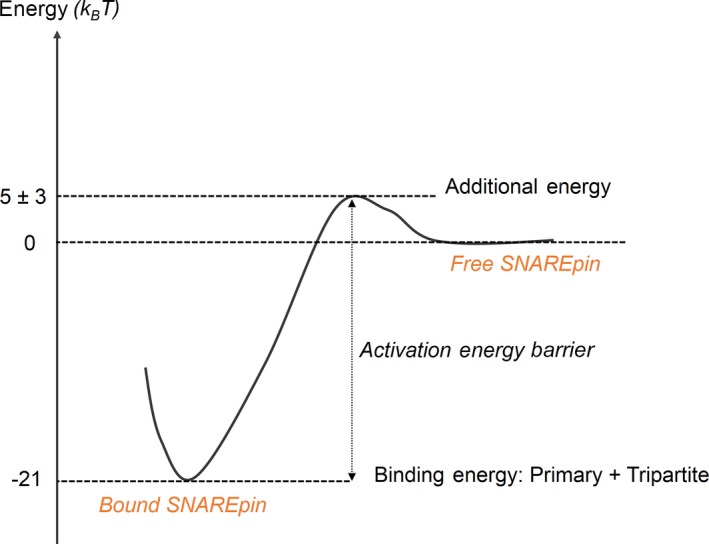
The SNAREpin can be in two states: bound to C2B or free. The energy of the free‐state is 0 and that of the bound state −21 *k*_B_ *T*. An additional energy barrier, Δ*E*_a_ = 5 ± 3 *k*_B_ *T* suffices to explain the observed spontaneous release rate.

If the primary Syt C2B were to remain bound to the SNAREpin during this dramatic re‐orientation, the new geometry thus created would most likely force the zippering (TMD‐linked) end of the SNAREpin away from the PM toward the SV in a highly implausible arrangement (Fig. A5). Therefore, it appears that the SNAREpin is most likely released from its primary Syt C2B during the Ca^2+^ triggered re‐orientation that disassembles the inner ring. Breaking the primary Syt site will cost ~ 13 *k*_B_ *T* 24, whereas the energy reward for Ca^2+^‐dependent loop insertion into the PM is ~ 21 *k*_B_ *T* 59, so overall loop insertion will be favored in a coupled process. In addition, the zippering force (locally up to as much as ~ 100 pN; Appendix 1) will help to actively strip the C2B off of the SNAREpin. Based on this, we anticipate that the SNAREpin is simultaneously released from the primary Syt as it re‐orients upon binding Ca^2+^. This would also render the assembled cis‐SNARE complex free for postfusion disassembly by SNAP and the NSF ATPase, enabling the v‐SNARE VAMP to be recycled along with the liberated Syt1 by endocytosis 8.

It seems likely, but not as certain, that the second, tripartite‐bound Syt C2B will similarly be released at some stage during the terminal zippering process prior to fusion, as suggested by Zhou *et al*. 24. From the consideration of our model, we also anticipate that the SNAREpin would need to release its tripartite C2B to complete fusion, as follows: The attachment of the tripartite C2B domain to the SV will strengthen after Ca^2+^ enters via aliphatic loop insertion, so it will remain bound to PS in the outer monolayer of the SV and simply translate inward radially along with its bound SNAREpin as the latter zippers up. However, modeling suggests that the sharp curvature of the SV will sterically limit the radial translation of the SNAREpin held in this orientation to a minimum radius of ~ 6.5 nm (as measured to end of fully zippered four‐helix bundle), whereas fusion can occur only at < 2 nm (Appendix 1). Ample energy is available to strip off the bound tripartite C2B (~ 8 *k*_B_ *T* 24) made available by zippering the final ~ 1/3rd of the four helix bundle from layers +5 to +8, which yields ~ 20 *k*_B_ *T* 18, 60.

The outer MUN ring may also contribute to clamping by binding the N‐terminal portion of the SNAREpins 28, and if so release would be correspondingly facilitated if the MUN ring were also disrupted by Ca^2+^. We note that the MUN domain is expected to angle away from the PM when its flanking C2B domain binds Ca^2+^ and rotates to insert in the PM (see fig. 9 in 27) a motion that would disrupt the co‐planar orientation with the PM needed for a ring.

Another interesting point concerns the fate of the six synaptophysin molecules that we propose nucleate the v‐SNARE (VAMP) proteins that form the SNAREpins from the SV. Synaptophysin binding to the VAMP TMDs within the SV bilayer will likely restrain them from zippering with their cognate syntaxin TMDs 61 as the fusion pore opens. If so, synaptophysin could function as a clamp within the SV membrane that could stabilize intermediates to controls the kinetics of fusion pore formation, expansion, and/or kiss‐and‐run behavior 62.

## Possible molecular origins of the primary and tripartite Syt C2B domains

The inner ring of ~ 18 C2B units is expected to assemble from Syt1 (or analogous ‘fast‐acting’ Syts such as Syt2 and Syt9) which are present in SVs at 15–20 copies per vesicle. It is likely that the ring is preformed at the SV before the vesicle contacts the PM, because isolated Syt C2B domains will form ring oligomers in solution triggered by ATP at its physiological concentration in the cytoplasm 22. These rings efficiently transfer from solution to lipid monolayers containing PIP2, because binding of the ‘poly‐basic’ site of C2B to PIP2 (10–15 *k*_B_ *T*) is much stronger than to ATP (4 *k*_B_ *T*) 23.

Based on this, we expect that preformed ~ 25 nm diameter Syt1 C2B rings emanating from the SV will immediately seal onto the Syntaxin‐rich ~ 75 nm diameter nano‐domains that self‐organize by binding PIP2 on the inside surface of the PM and are thought to act as ‘molecular beacons’ for SV docking 10, 13, 22. Given their high local concentration in the SV (~ 10 mm on the SV surface) as compared to the low ~ 10 μm *K*_d_ for oligomer assembly 22 essentially all of the SV's supply of Syt1 (or similar) is likely to be utilized in forming a single ring before encountering the PM.

If this is the case, where do the additional C2B domains needed to occupy the tripartite binding sites come from? One possibility is that they come from an additional supply of Syt1 (or similar) that resides in the PM 63. But it is also possible that they derive from a different class of primarily PM‐localized Syt, such as Syt7, or cytosol‐derived Doc2 that have much higher intrinsic affinities (in absence of membranes) for Ca^2+^ (< 10 μm) than Syt1 (or similar; > 100 μm).

Syt7 is the best studied of these high affinity sensors and is found in the PM not the SV 63, 64. Syt7 has been functionally linked to ‘asynchronous’ release, in which SV fusion is triggered by low 0.5–2 μm Ca^2+^ levels that can persist for up to several hundreds of ms after voltage‐gated Ca^2+^ channels have closed, well after ‘synchronous release’, which keeps close pace with the triggering action potential and occurs within the very short (ms) period during which these channels are open 20, 63, 64. Synchronous release is mediated by Syt1 (and similar) which because of their much lower intrinsic affinity for Ca^2+^ do not trigger SV fusion except at peak local Ca^2+^ concentration (20–100 μm 20, 65) and they become ineffective right away when Ca^2+^ is rapidly reduced below this peak.

Interestingly, the C2B domains of Syt1 (and similar) are all able to bind both the primary and tripartite sites on the SNAREpin‐Cpx complex based on conserved sequences 24. By contrast, the C2B domain of Syt7 (and similar; and all C2A domains of Syts) lacks the sequences needed for primary SNAREpin binding to SNAP‐25, but they all contain the helical extension 24 that mediates tripartite binding to Cpx, VAMP, and Syntaxin. As a result, the slow/high affinity Ca^2+^ sensors (Syt7 and similar) are only able to target the SNAREpin at the tripartite site, while the fast/low Ca^2+^ affinity sensors (Syt1 and similar) can target both SNAREpin sites. Since the SV's supply of Syt1 is likely consumed (into a preformed ring) even before it docks to the PM, it seems likely that the tripartite C2Bs may derive in significant part from Syt7 (or similar high affinity/slow‐acting Ca^2+^ sensors) emanating from the PM rather than the SV. It is also worth noting that the tripartite C2B must be added to the structure after the initial SNARE assembly because Cpx, which is required for the tripartite interaction, can only enter after its own binding site at the VAMP‐Syntaxin interface has been formed 53.

In short, Syt7 (or similar) has the potential to form the upper half of the vice that clamps the SNAREpin in place with Syt1 (or similar) constituting its lower half (Fig. 3A). The top (Syt7) layer of this protein sandwich would then have a much higher affinity for Ca^2+^ than the bottom (Syt1) layer, allowing each type of Syt to play a distinct role in clamping and release. This arrangement raises new possibilities that could explain how both synchronous transmission (release of the lower, stronger ring clamp at high Ca^2+^) and asynchronous transmission (release of the upper, weaker buttressing clamp at low Ca^2+^) could occur stochastically from the same population of primed SV.

It is premature to suggest whether and under what circumstances the upper and lower clamps are independent of each other (i.e. both clamps are needed to restrain SNAREpins so that releasing either one releases SNAREpins) or contingent on each other (i.e. either clamp is sufficient so that both clamps need to be released to release SNAREpins). Either way, the buttressed‐ring model suggests novel ways of re‐interpreting existing physiological and genetic data and for formulating experimental approaches. For example, it is noteworthy in terms of this model that while Ca^2+^ binding to the Syt1 C2B domain is critical for synchronous release, it is rather the reverse for Syt7: its C2A domain must bind Ca^2+^ to enable asynchronous release 61 and Syt7's C2A far dominates over its C2B domain in membrane‐binding affinity and the ability to sandwich between two negatively charged membranes in its Ca^2+^‐bound state 66. One can readily imagine that in the process of moving to closely adhere the SV to the PM that the proposed upper Syt7 C2B clamp (tripartite site) could be released.

## Other variations on the buttressed‐ring hypothesis

It is important to state that for purposes of clear exposition, we have described only the simplest version of our hypothesis. For example, we have supposed that the outer MUN rings are stable and produce exactly six SNAREpins in a concerted reaction. But it could also be that MUN domains do not assemble into stable rings but rather that individual Munc13 units come and go in the same planar geometry we propose forming transient or partial rings. In this sequential, stochastic variation of our model, individual MUN domains snugly but dynamically approximate the stable Syt ring to template and load a SNAREpin potentially anywhere onto the inner Syt1 ring, and not just rigidly at every 3rd position of the inner ring as the stable MUN ring model would require. Theoretically sequential interactions could result in an inner ring in which every Syt C2B is loaded with a SNAREpin, since according to our structural model (Fig. 4C) neighboring SNAREpins on the Syt ring do not clash (as long as they do not zipper beyond layer +4). However, once the first SNAREpin has been templated, it will pull the SV toward the PM until it closely approximates the surface of the SV against the inner ring, sharply reducing steric access needed in the stochastic model for subsequent SNAREpin assembly to take place. For this reason, we favor the concerted mechanism. However, something in between the two extremes would relax the strong constraint in the stable outer ring model on Syt ring size, requiring 18 C2Bs to fit within a stable ring of six MUN domains.

It is also likely that although the inner ring can be stable as an overall entity, individual bonds joining the C2Bs may dynamically break and re‐form, and complete rings may not always be present, or even predominate, depending on the conditions. For that matter, even very short oligomers of Syt1 C2Bs could also serve to clamp SNAREpins by restraining them from rotating away from the vertical orientation in which terminal zippering is sterically prevented. Modeling suggests that even individual SNAREpins (in the vertical arrangement with their bound C2Bs attached to the membranes below and above) would be restricted from inward radial movement; but without the lateral restraints due to primary C2B oligomerization, they would be more prone to tilt (enabling zippering) and could not be synchronously released.

Another important unknown concerns the disposition of the C2A domains. Each Syt has a membrane‐proximal C2A domain separated from its membrane‐distal C2B domain by a flexible linker region 24, 67. The C2A domain of Syt1 is located flexibly outside the C2B ring 21, and there is ample room in our proposed structure to accommodate these domains similarly in our model (Fig. 3A). It will be especially important to learn how the C2A domain of Syt7 is positioned before and after it binds Ca^2+^ because this event is critical for triggering asynchronous release and dominates over its C2B domain 63.

Finally, generalizing beyond exocytosis, SNARE‐dependent fusion enables vesicle traffic throughout the cell 1, 68. These ‘constitutive’ fusion processes are not linked to Ca^2+^ and do not involve Syt or Cpx. They all, however, require one or another MUN domain‐containing protein (which are typically termed ‘tethers’) 69 but which clearly also template SNARE assembly as a core aspect of their biochemistry 15, 24, 28. Given the conserved size and sequence of MUN domains, we suggest that MUN‐containing proteins may generally assemble into hexameric rings that co‐operate with an SM protein (analogous to Munc18) to template SNAREpins. In such cases, there will be no inner Syt ring or Syt buttress to the SV to clamp the SNAREpins, so fusion will follow constitutively. This extension of our hypothesis suggests that each constitutive fusion event will involve only a handful of SNARE complexes and associated stoichiometric MUN‐containing chaperones as recently found by super‐resolution imaging of constitutive fusion at the PM 70.

## Quantitative considerations

Returning to Fig. 3A, is the proposed Syt C2B inner ring physically capable of preventing fusion? We examine this from the perspective of soft matter physics, which has the simplifying advantage of focusing on overall material properties as distinct from details of chemistry. Rand and Parsegian 47 discovered that lipid bilayers abruptly destabilize when the hydrostatic pressure is raised above a critical pressure. This happens when their surfaces are dehydrated beyond a critical point, tipping the energetic balance in favor of radically curved nonbilayer structures (such as inverted hexagonal phases, rhombohedral phases) 71, 72. An analogous abrupt transition is also observed when two bilayers are directly pressured together in the surfaces forces apparatus 73. Following on this pioneering work, detailed measurements suggest that the transition pressure is in the range of 200–500 atm (one atm is the typical pressure of the atmosphere at sea level) for lipid compositions bracketing the inner leaflets of the SV and PM (see Appendix 1 for details) 48, 49.

A symmetrical structure like a ring will mechanically distribute the force load due to the SNAREpins much as a well‐designed building distributes the load it bears due to gravity. This helps minimize the pressure. The hydrostatic pressure due to six SNAREpins bearing on a ring of 25 nm diameter is at most 30 atm (Appendix 1) far below the transition pressure range for fusion. Clearly, the ring is physically competent to prevent fusion – at this low pressure, spontaneous fusion would only occur over days (Appendix 1).

However, when the ring disassembles upon binding Ca^2+^, this situation drastically changes. The liberated SNAREpins and the opposed bilayers are both now free to move inward in a coupled fashion. As they get closer, they exert their force over an ever‐smaller area, and pressure rises geometrically to reach 400–500 atm when their zippering tips circumscribe a disc of ~ 4 nm diameter (Appendix 1). This will create an initial fusion pore opening no larger than ~ 2 nm in diameter consistent with experimental observations 55, 58. This should require < 1 ms (Appendix 2). The SNAREpins will need to rotate in the plane to point inwards radially and simultaneously translate inward pulled more rapidly than diffusion by their own force against the viscous bilayer until they reach the critical separation of ~ 4 nm (taking ~ 20 μs). SNAREpin zippering brings the inter‐membrane distance below 1 nm whereupon fusion spontaneously occurs (taking ~ 10 μs up to but not including bilayer coalescence). Finally, the two bilayers destabilize and merge (likely via a hemi‐fusion intermediate) as determined by the time required to for two thin and closely applied viscous fluid‐like bilayers to coalesce under pressure (taking < 1 μs). This process may be further accelerated by Ca^2+^‐binding loop insertion of Syt C2B, and other physical factors which may lower membrane tension 74.

Upstream of this, de‐clamping (i.e. liberating SNAREpins after entry of Ca^2+^), is likely to be slower. Even though a precise time range cannot be rigorously estimated from currently available information, some insights are possible (Appendix 2). The rate of inner ring disassembly will be determined by the rates of Ca^2+^ binding, primary Syt1 C2B re‐orientation and loop insertion into the PM. When unconstrained by a bound SNAREpin, this occurs experimentally in the sub‐millisecond time domain 75. But as we have already noted geometry requires that re‐orientation and loop insertion are at some stage linked to the removal of C2B from the primary site on the SNAREpin (Fig. A5), a bond of ~ 13 *k*_B_ *T* that will spontaneously dissociate no faster than 30–3000 s^−1^ far too slow to enable the fusion pore to open synchronously in < 1 ms. A likely explanation is that the primary C2B is removed far faster by applied force than according to its intrinsic dissociation rate. For example, the sterically coupled loop insertion should produce a force in the range of 10–100 pN (10–30 *k*_B_ *T* released over 1–3 nm; see Appendix 1). By contrast, the lower affinity of tripartite C2B (which we suggest is derived from Syt7) binding should not impose a kinetic impediment (~ 8 *k*_B_ *T*; spontaneous rate of dissociation between 1000 and 100 000 s^−1^). Taken together, these considerations make it plausible that our model is consistent with the observed time required from binding of Ca^2+^ to opening of the fusion pore of the 0.3–0.5 ms for evoked synchronous quantal release 76.

In contrast to the high rate of evoked release, synapses require an extraordinarily low rate of ‘spontaneous release’ (release in the absence of an action potential) to suppress background noise in the brain to enable single vesicle sensitivity. The probability of spontaneous fusion of a docked vesicle (in the readily releasable pool) is reported to be 0.002–0.006 per second per vesicle (about one in 200 each second) 77, 78, 79, 80. Release in the absence of an action potential can in principal either be intrinsically spontaneous or alternatively it can be triggered by a spontaneous rise in local Ca^2+^. The latter mechanism can account for much (30–60%) of such action‐potential‐independent events 77, but for purposes of this order‐of‐magnitude calculation, we can ignore the Ca^2+^‐dependent component. In our model, there are six clamped SNAREpins per vesicle, then because a single SNAREpin can efficiently drive fusion 55, 81, each SNAREpin would have to have a probability of about 1/1000 of spontaneously slipping out of its clamp each second. In the simplest model, this escape rate would result from overcoming an activation energy in the range of 26 ± 3 *k*_B_ *T* (Appendix 3). In our model, the clamp is composed of a SNAREpin's primary (~ 13 *k*_B_ *T*) and tripartite (~ 8 *k*_B_ *T*) C2B domains buttressed by the C‐terminal domain of Cpx that grips the SV (~ 12 *k*_B_ *T* for AH and CT subdomains combined 82) and potentially also by the MUN outer ring. We conclude that the energetics of the proposed clamping mechanism predicted from our model are plausibly in accordance with the measured rates of spontaneous release.

## Perspective and open questions

We see that C2 domains are rigid yet versatile protein modules that control reactions by changing orientation on and between membranes, and they are extensively utilized to orchestrate precise release of neurotransmitters by SNARE proteins at neuronal synapses.

It must be clearly stated that ring structures such as we propose (be they stable or dynamic) have not yet been directly observed either *in vivo* or in isolated systems capable of partly replicating synchronous neurotransmitter release 70, 83, 84, 85. To date, however, the methods applied would not be expected to reveal these structures without intentional effort were they present and none of the cell‐free systems yet utilized have the complete set of proteins that are required or have been documented to actually achieve sub‐ms fusion following addition of Ca^2+^. It is also possible that partial or dynamic ring oligomers may be especially labile precisely because of the highly stressed metastable state in which they must exist to function. In that case, visualizing the buttressed rings would require special genetic, physiologic, or biochemical conditions to preserve them to favor their observation.

Our speculative ‘buttressed‐ring hypothesis’ affords many specific and testable structural, biochemical, and physiological predictions and equally raises even more open questions, some of which we have explicitly mentioned. We believe that the hypothesis provides a needed integrated, framework from which to view this complex field, even at the risk of some oversimplification. By focusing attention on what we feel are the key outstanding questions we hope that our model will productively facilitate discussion and encourage new research directions. Like most hypotheses when they are first put forward, it seems likely that not all details as currently outlined will ultimately be correct. But like every good hypothesis, it may stimulate a faster approximation to a deeper understanding, in this case of one of the central problems in neuroscience and in cell biology.

## Author contributions

JER, SSK and FP: Conception, data analysis, drafting and revising the manuscript. KG: Data analysis, drafting manuscript.

## Appendix 1 Novel soft matter ‘Pressure Model’ for membrane fusion and its implications concerning the stability of the clamp

The purpose of this Appendix is to establish whether the proposed ring structure is physically competent to clamp SNARE‐dependent membrane fusion, and then to dissect the transition from the clamped to the fused state into several sequential steps. Our results illustrate how a simple physics model can predict a time constant for fusion consistent with the physiological one.

Zippering of the SNAREpins brings the membranes progressively closer. There is negligible resistance to this movement until the membranes are ~ 2 nm apart 47. Below that distance, the membranes effectively repel each other through a combination of short‐range repulsion due to bound water that has to be removed (hydration forces) and to the vertical bobbing movements of bilayer lipids (protrusion forces). These forces were well characterized in the 1980s by Rand and Parsegian 47. Since both forces are proportional to the interacting membrane area, they may be treated as pressures (i.e. force divided by the area over which it is applied). Empirically, it has been determined that the overall pressure, *P*, decays exponentially with the thickness of the water layer between the bilayers *d*:* P*(*d*) = *P*_0_exp(−*d*/λ). Typically, *P*_0_ = 10 000 atm and λ = 0.2 nm 47 (Fig. A1A). It was observed that when the membranes are sufficiently dehydrated, they are no longer stable as parallel/lamellar bilayer structures and abruptly transition to nonbilayer phases (such as inverted hexagonal phases, rhombohedral phases) at a critical transition pressure 71, 72). This transition occurs when *d* is below ~ 0.5–1 nm, which corresponds to a pressure in the range 200–1000 atm 47, 49, 86 the exact value of which depends on bilayer composition. Biologically relevant compositions containing phosphatidylethanolamine (PE) are at the lower end of the range, whereas, for example, pure phosphatidylcholine (PC) is at the higher end of the range. As this suggests, the transition pressure is usually larger for membranes made of pure lipids and decreases in the presence of lipids which have an intrinsic conical shape. It is also worth noting that cholesterol, even at 50% per mole, does not significantly affect the overall transition pressure 47, 87. Finally, the presence of charged lipids can modify the short‐range repulsion; for instance, when there are charged lipids, the addition of Ca^2+^ (at concentrations > 1 mm which are unlikely to be physiologically relevant) can significantly reduce the transition pressure and facilitate fusion 88, 89.

It is well recognized that a transition to nonlamellar phases resembles what happens when two membranes are merging 46, 90. Hence, for fusion to occur, the short‐range repulsions have to be overcome and the membranes need to be at distances below 1 nm from each other where they are too dehydrated to be stable. The activation energy barrier that must be overcome for the bilayers to fuse is, therefore, equal to the work required to overcome the short‐range repulsion. The activation energy and the transition pressure and distance all depend on the lipid composition. Luckily, a model composition reasonably resembling the cytoplasmic leaflets of the SV 9 and the PM 91, 92 has been well characterized 48, 49, consisting of dioleoylphosphatidylcholine‐dioleoylphosphatidylethanolamine (DOPC/DOPE, 60/40). The corresponding short‐range repulsion profile is presented in Fig. A1A. The transition pressure and distance are 440 atm and 0.6 nm, respectively. The activation energy has also been measured and is ~ 35 *k*_B_ *T* 93. This same energy value will also be obtained by integrating the pressure (Fig. A1A) that needs to be applied to overcome short‐range repulsion to transit the bilayers from infinite separation to the transition separation of 0.6 nm when this value is multiplied by the surface area over which the pressure needs to be applied. For this composition, we calculate that this area is 13 nm², corresponding to a disk of about 4 nm diameter. Physical‐chemically this surface area is occupied by about 20 phospholipids on each surface of the contacting bilayers and represents the minimum number of lipids in each leaflet needed to nucleate the fusion pore for this lipid composition. Hence, it is the minimum area over which the ~ 400 atm transition pressure must be applied to trigger fusion.

Given enough time, thermal fluctuations will overcome any level of activation energy resulting in spontaneous fusion. However, the larger the activation energy, the longer this will take. It has been shown in Kramer's theory 94, 95 that this waiting time, τ, increases exponentially with the activation energy according to the equation, τ = τ_0_exp(*E*_a_/*k*_B_ *T*). For reactions that occur over ~ 2 nm, the prefactor τ_0_ has lower and upper limits of 10^−9^–10^−7^ s 96. For an activation energy of 35 *k*_B_ *T*, the waiting time will be between 3 weeks and 5 years, explaining why spontaneous fusion of membranes does not happen on physiological timescales.

As they zipper SNAREpins mechanically pull the bilayers inward toward each other, providing the source of the pressure to overcome the short‐range repulsive forces. When enough energy has been provided to exactly overcome the activation energy, then fusion will occur. For the composition we are considering, this will require exerting at least 440 atm over a disk of 4 nm diameter or more and will occur when the membrane separation is reduced to 0.6 nm. From the force exerted by each SNAREpin and the number of pins, we can calculate the pressure they exert upon the disk circumscribed by their zippering ends as a function of the separation between these ends.

The force produced by each SNAREpin can be reasonably estimated using the previously measured energy minima and maximum of zippering of the last layers of the SNAREpin 18. The actual position of these energy extrema during SNAREpin zippering is unknown, but we know that they include the last five hydrophobic layers of the coiled‐coil (+3 to +8) and the linker domains. Based on the overall length of the fully assembled four helix bundle (12 nm), ~ 5 nm for the zippering of the last stretch seems reasonable. The resulting harmonic energy landscape is shown in Fig. A1D. The force is then the derivative of the energy landscape, which, with this model, peaks a little over 100 pN at 1.2‐nm inter‐membrane distance, which corresponds to the zippering of the linker domains of Syntaxin and VAMP2. Also, 100 pN is consistent with detachment forces of the SNAREpin measured by atomic force microscopy 97. Hence, it is realistic to choose 100 pN as an upper bound for the force. Assuming that there are six SNAREpins that each pull on the membrane with a 100 pN force, the maximum total force is *F*_max_ ~ 100 pN.

To estimate the area over which this force is applied, the geometry of the SNAREpins in the Syt1 ring model has to be detailed (Fig. A1B). The outer and inner diameters of the 18 subunit ring are 25 nm and 19 nm, respectively. Layer +1 is located above the ‘mid‐ring’ with a diameter *d* = 22 nm and the SNAREpins extend inward over a distance *L* = 7 nm with an angle, α = 40° from the mid‐ring (Fig. A1B). Because they are bound to primary C2Bs, they cannot rotate or move toward the center of the ring. At this fixed position, they will zipper as much as possible and pull the membranes which will deform as presented in Fig. A1C. The contact area is taken to be that of a disk with a diameter (*s*_0_) equal to the tip‐to‐tip distance between two facing SNAREpins (Fig. A1B). In our ring model, $s_{0}=\sqrt{d^{2}-4Ld\text{sin}\left(\alpha \right)+4L^{2}}$  = 17 nm, which corresponds to an area of: *A*_clamp_ = 225 nm².

The resulting pressure, *P*_clamp_, can be directly calculated: *P*_clamp_ = *F*_max_/*A*_clamp_ = 6 × 10^−10^/225 nm² ~ 30 atm which is much below the 440 atm threshold (and indeed far below the entire range of transition pressures for all compositions). The corresponding reduction in the activation energy (Fig. A1A) is Δ*E*_a_ = 2.2 *k*_B_ *T*. Hence, applying Kramers’ theory, upon the action of the SNAREpins bound to the Syt ring, fusion will occur with a characteristic time of typically τ_0_exp((*E*_a_ − Δ*E*_a_)/*k*_B_ *T*) which is 2 days to 6 months. This shows that SNAREpins will not be able to induce fusion on a reasonable timescale when they are held apart and positioned in the geometry expected from the structural biology incorporated into our model. In addition to these considerations, the thickness of the ring (about 4.5 nm) keeps the bilayers separated well beyond the inter‐membrane separation of 0.6 nm to trigger fusion of this lipid composition.

Conversely, when they are released, the SNAREpins must be able to fuse the bilayers. In this simplest analysis, we assume that all six of the SNAREpins move collectively toward the center. As they gather, the pressure they exert progressively increases because a constant total force is applied over an ever‐smaller area. After the SNAREpins are located close enough to the center, they bring the membranes in sufficiently close vicinity to dehydrate them and induce fusion. Then, the two membranes start merging. Note that there is likely mechanical cooperativity among the SNAREpins because they are coupled via the membranes into a single system; this will further facilitate fusion.

To check whether this can realistically be achieved, we have to analyze the energy landscape resulting from the addition of the inter‐membrane short‐range repulsion and the force applied by the six SNAREpins over the minimum area over which pressure must be applied, 13 nm². The resulting curve is presented in Fig. A1E. For the harmonic energy landscape in Fig. A2D, SNAREpins are not able to fully balance the short‐range repulsion due to the DOPC/DOPE (60/40) membranes. An energy minimum is reached at 0.7 nm inter‐membrane distance. According to this landscape, the remaining activation energy for fusion (needed to force the bilayers closer to achieve the 0.6‐nm critical separation to trigger fusion) is 4.6 *k*_B_ *T*. Using Kramers’ theory, we conclude that this activation energy is spontaneously overcome by thermal fluctuations in 0.1 μs (lower bound) to 10 μs (upper bound).

How much time is required for the bilayers to merge once the transition pressure occurs? Once again we can turn to soft matter physics by modeling fusion as the pressure‐driven coalescence of two closely apposed, viscous materials, which as we will see should occur in the range of 1–10 μs. We have just seen that the time required for thermal fluctuations to bring the two membranes to the transition separation (< 1 nm) ranges between 0.1 and 10 μs. After the membranes have passed the activation energy barrier (Fig. A1E), the movement becomes diffusion‐limited and results from a combination of changes in the relative positions of the SV and the PM and/or the shape deformations of either. Deformation of the membrane is difficult to model but would only accelerate fusion. The rate of movement of the SV and PM toward each other (Fig. A2) is at steady‐state when the zippering force is balanced by opposing the viscous drag. Because the viscosity of lipid greatly exceeds that of water, the membranes limit the rate. We use a safe upper bound for the lipid viscosity, 1 Pa.s., which is 1000 times larger than the viscosity of water. Again, to be certain we will calculate an upper bound of the merging time, we will assume that the viscous force acts over the whole cylinder that encompasses the overlapping regions of the two membranes (radius *r*_fus_ = 15 nm and thickness δ = 5 nm; Fig. A2). The viscous force when moving an object of this radius at speed *v* is the Stokes force: 6π*r*_fus_η*v* that has to be balanced by the (assumed) constant force coming from six SNAREpins zippering the end of their linker region and the beginning of their transmembrane domains. Since we want an upper bound for the membrane merging time, we will take the lower bound for the unknown force exerted by each SNAREpin to be *F*_trans_ = 10 pN. Then, the total force is 6*F*_trans_ = 60 pN. Using the Einstein equation, one gets: *v* = *F*_trans_/(π*r*_fus_η), from which the time to move over δ is π*r*_fus_ηδ/*F* = 0.4 μs. This means that merging of the membranes is instantaneous after the SNAREpins have all migrated toward the center.

Clearly, these calculations can only result in estimates because they involve many assumptions including the energy landscape of the SNAREs and a specific membrane composition. However, in every case, we have been careful to make upper limit calculations that disfavor our hypothesis. Yet, they show that a simple pressure model provides an explanation at the level of soft matter physics for sub‐millisecond SNARE‐induced fusion following release of the clamp. Note that unlike more granular models of fusion 98, 99, 100 that concern themselves with particular alternative arrangements of lipids in the transition state, the pressure model takes a system‐level approach that avoids these complexities while remaining consistent with the various detailed molecular proposals.

Additional notes:

- The pressure/energy view is valid only when a sufficient number of SNAREpins are involved and delimit a clear area over which the pulling force is applied. When a small number of SNAREpins (1, 2, and maybe 3) are triggering the fusion process, the concept of pressure cannot be used because it becomes difficult to define an area, and alternate views are more appropriate. For instance, approaching the membrane in very close vicinity increases the collision frequency of the membranes which lowers the prefactor τ_0_ in Kramers’ theory and accelerates the process.
- The pressure is always at equilibrium on the timescale of the fusion process because it equilibrates at the speed of sound (~ 1500 m·s^−1^), that is, within ~ 10 ps for a 10‐nm range.
- We assume above that all of the force from SNAREpin zippering is directed perpendicularly inward toward the membrane planes. There will, of course, be a component of force that is directed radially inward. But it is reasonable to ignore this when modeling the pressure close to the transition because the transmembrane domains are increasingly perpendicular to the membranes as the SNAREpins complete their zippering, the zippering force is increasingly fully directed toward fusion.

## Appendix 2 Concerning the time required for the clamp to be released and SNAREpins to migrate to achieve the critical transition pressure for fusion

Appendix 1 did not consider the time required to release the clamp after Ca^2+^ rises locally to a concentration that can trigger synchronous vesicle release (> 20 μm). We do so here by making a few reasonable assumptions and conclude that these steps will occur in < 25 μs.

As indicated in the main text (under Quantitative Considerations), two steps will be considered after Ca^2+^ binding and breakage of the C2B‐C2B bonds: (a) Rotation of the primary C2B in water and insertion of its Ca^2+^‐binding site aliphatic loop residues into the PM bilayer; and (b) rotation and inward radial translation of the bound SNAREpins under their own force through the viscous bilayer to reach a tip‐to‐tip separation needed to reach the transition pressure range (~ 4 nm) as follows:

- Rotation of C2B in water and insertion of its Ca^2+^‐site loops into the PM bilayer (< 20 μs). Once C2B is released from the ring, it can freely rotate and the loop will be positioned to insert in the membrane. *A priori*, the only force that opposes this rotation is viscosity. Thus, this movement is diffusion‐limited. In reality, in addition to thermal fluctuations, the zippering of the SNAREpin will facilitate the rotation. This means that calculating the characteristic time of rotation by diffusion will provide an upper bound for the actual value. Upon thermal fluctuations, the protein will stochastically rotate with an instantaneous angular velocity ω. Since this rotation is actually driven by SNAREpin zippering, it will be oriented and we will only consider rotation along the corresponding most favorable axis. C2B will be modeled as a cylinder of diameter *e* and length *l* (Fig. A3A). Because of the viscosity of the aqueous medium, η, there is a viscous torque that opposes the movement. This torque, *M*, resembles the one that has to be applied in a Couette viscometer: *M* = π*e* ^2^ηω*l*. Using this expression, the tangential friction force is *F* = *M*/*e* = π*e*ηω*l*. When the cylinder is rotating, the speed of the surface is *v* = ω*e*/2. The rotational diffusion coefficient can then be deduced from the Einstein relation: *D* = *vk*_B_ *T*/*F* = *k*_B_ *T*/(2πη*l*). In standard diffusion, the average total displacement is related to the diffusion coefficient and the displacement by $\delta =\sqrt{Dt}$. Here, δ is the distance traveled by the surface of the cylinder during a 90° rotation, that is, a $\delta =\left(\frac{\pi e}{4}\right)$. The characteristic time to rotate C2B is $\frac{{\left(\frac{\pi e}{4}\right)}^{2}}{D}=\frac{\pi^{3}e^{2}\text{ln}}{8k_{B}T}$ << 1 μs assuming *e* = *l* = 5 nm and η = 10^−3 ^Pa.s, that is, the viscosity of water. Hence, this rotation process can be considered instantaneous (1 μs) in relation to the timescales of the other steps involved.

After the Ca^2+^ loops are correctly oriented by rotation of the C2B, they can insert into the membrane. The shape of the loops resembles a cylinder with a 2.5‐nm diameter circular section that inserts vertically 1 nm inside the inner leaflet (Fig. A3B). The insertion of this cylinder is considered to occur in two steps. First, the hydrophilic layer coming from the polar heads of the lipids forming the inner leaflet of the PM must be moved away so that the hydrophobic loops can begin to contact the hydrophobic core of the bilayer. Second, the loops have to insert into this transient hole to fill it.

This is equivalent to placing a hydrophobic defect in the membrane right next to the loops. In multicomponent membranes, such defects spontaneously occur and typically cover 5% of the membrane surface at any one time 101 meaning that 5% of the membrane surface is hydrophobic. Because lipids cover 1 nm² in 100 ns by diffusion 102, 103 and the circular section of the cylinder facing the membrane is 5 nm² (= π·1.25²), a hydrophobic defect will have passed below any such C2B loop in 10 μs ((5 nm²·100 ns·nm^−^²)/5%). It is reasonable to assume that this is the same as the time required for removing the polar heads initially located below the loops.

Once the hydrophobic loops attach to the surface of a suitably sized defect, they then need to move in to fill it. In order to obtain an upper bound estimate of the characteristic time for loop insertion, we will assume that there is no force other than viscous forces to oppose this insertion into the PM, that is, we assume that loop insertion at this last step is diffusion limited. In reality, active attractive hydrophobic and electrostatic forces between the loops and the PM drive and accelerate this insertion. Since we do not know the forces, we ignore them to once again bias our estimate to an upper limit.

Because of the Stokes force, the diffusion coefficient is inversely proportional to the circular cross‐section of the cylinder. Hence, the relevant section is the one that penetrates vertically inside the hydrophobic layer, which is 5 nm². This is to be compared to the section perpendicular to the lateral movement of a protein with a single transmembrane domain like SNAREs; this section spans both hydrophobic leaflets (5 nm) and has a width of ~ 1 nm which means it covers 5 nm². Thus, the diffusion coefficient of the loops will be similar to that is of individual SNAREs which is *D* ~ 1 μm²/s 104. Using this value for the diffusion coefficient of the loops will provide a good approximation of the insertion time. Since the loops will vertically insert inside the inner leaflet to a depth, δ ~ 1 nm, the average time to cover this distance by diffusion is directly related to the diffusion coefficient and the displacement by δ²/*D* ~ 1 μs, which can also be considered instantaneous.

Based on these calculations, it is reasonable to assume that the total time for C2B rotation and loop insertion is < 20 μs.

- Rotation and inward radial translation of the bound SNAREpins through the viscous bilayer (5 μs): As explained in the main text (under Quantitative Consideration), during the rotation of the primary C2B unit and loop insertion, the bound SNAREpin will begin zippering. Initially, it will be located almost at the same position as in the ring structure. While zippering, it will exert a force/torque that will result in an effort to rotate and translate it toward the center of the ring. This force/torque also exists in the clamped state (Appendix 1), but the C2B/SNAREpin bonds provide the resistance against this force and prevent any movement. When released, the SNAREpin can freely move inward driven in by the force of its own zippering. As the available nonzippered part of the SNAREpin becomes shorter, the transmembrane domains are pulled toward smaller inter‐membrane distance. In this paragraph, we estimate the time it takes for the SNAREpin to move in to the critical separation to reach critical pressure for fusion.

Because the transmembrane domains have to remain inside the membranes, the effective force, *f*, that contributes to the movement is the projection of the total force, *F*, tangentially to the membranes (Fig. A4): *f* = *F*cosβ, where β is the angle between the membrane and the direction of the force. Because the system is in a viscous regime, the equation of motion is obtained by the Einstein relation: *v* = *fD*/*k*_B_ *T*,* D* being the diffusion coefficient of the SNAREpin. The average zippering force of the last layers of the SNAREpin is obtained from the energy landscape in Fig. A1D, ~ 30 *k*_B_ *T* are released over 3 nm, that is, *F* ~ 40 pN and *D* ~ 1 μm²·s^−1^ 104. The initial angle depends on the exact location of the transmembrane domains but is at most β_init_ = 30°. Assuming the movement is almost linear in the top view projection, the equation of motion becomes: *v* = d*x*/d*t* = (*FD*/*k*_B_ *T*)cos(*x*/*R*‐π/6) where *x* is the displacement along the membrane and *R* is the radius of the vesicle. The time for SNAREpin rotation and inward translation is obtained by integrating this equation along a straight line (Fig. A4) from *x*_init_ = 0 (initial state) to *x*_fin_ = π*R*/6 (SNAREpin at the center), that is, *k*_B_ *T*·*R*/(*FD*(ln((1 + sin(π/6))/cos(π/6)) = 5 μs with *R* = 20 nm. Fusion will occur at least 2 nm before the transmembrane domain actually reaches the center of the ring (4 nm tip‐to‐tip separation used in Appendix 1).

To summarize our calculations in Appendices 1 and 2, after the C2B‐C2B bonds that stabilize the ring have been released, the complete fusion process will occur in < 50 μs (25 μs for C2B rotation and loop insertion +5 μs for SNAREpin inward movement and rotation +20 μs for fusion – see Appendix 1).

Additional note: These timescales do not require that a complete ring is formed. However, one of the merits of the ring, even partially formed, is that it promotes synchronicity by facilitating collective action of the SNAREpins. Indeed, several SNAREpins are prepared in the exact same primed state and released at the same time; since the complete molecular rearrangements that lead to membrane merging after Ca^2+^ binding is extremely fast in regard to the complete fusion process, all SNAREpins will simultaneously reach the 2‐nm radius circle where they will produce fusion.

## Appendix 3 Concerning the rate of spontaneous release

Spontaneous fusion will occur if even a single SNAREpin is released from its clamp 105. This happens at a rate of 0.012–0.033 s^−1^ at a typical central synaptic bouton 77, 78, 79, 80. Considering there are ~ 5 release ready (‘primed’) SV per bouton and, in our model, six SNAREpins per SV, the rate of spontaneous release of a single SNAREpin from its clamp is estimated to be 4 × 10^−4^–1.1 × 10^−3^ s^‐1^. SNAREpins are held by two C2B subunits and sandwiched between two membranes. In energetic terms, each SNAREpin exists in an energy well that it can escape from (to be released) as a result of thermal fluctuations that impart, to them an activation energy that exceeds the height of the barrier above the well. As in Appendix 1, this process can be modeled by Kramers’ theory. Knowing the rate of spontaneous release of one SNAREpin, it is then possible to work backwards to deduce the required activation energy, *E*_a_, through Kramers’ theory, *t*_s_ = τ_0_exp(*E*_a_/*k*_B_ *T*), where *t*_s_ is the slipping time (inverse of the rate of spontaneous release) and the prefactor τ_0_ is between 10^−7^ and 10^−9 ^s (as in Appendix 1). Hence, to reach the observed spontaneous release, 4 × 10^−4^–1.1 × 10^−3^ s^−1^, the required activation energy (*E*_a_) should be equal to 26 ± 3 *k*_B_ *T*.

Is this value reasonable in light of the binding energies involved in the structural model for the clamp? As shown in Fig. A5, the activation energy must be larger than the binding energies of SNAREpin to the Syt molecules, that is, larger than the sum of the energies of the bonds to both the primary (~ 13 *k*_B_ *T*) and tripartite C2Bs (~ 8 *k*_B_ *T*; energies estimated from the binding constants in 24), that is, larger than 21 *k*_B_ *T*. This value is reasonably consistent with the rate of spontaneous release but requires either additional stabilization (potentially via binding to the outer MUN ring) or an additional activation energy barrier, or both, combing to 5 ± 3 *k*_B_ *T*. An additional activation energy barrier is expected from the need for small molecular displacements required to effect the liberation of the pin from its vice.
